# New insights into the deactivation mechanism of V_2_O_5_-WO_3_/TiO_2_ catalyst during selective catalytic reduction of NO with NH_3_: synergies between arsenic and potassium species†

**DOI:** 10.1039/c9ra07545c

**Published:** 2019-11-19

**Authors:** Lin Li, Lin Chen, Ming Kong, Qingcai Liu, Shan Ren

**Affiliations:** Engineering Research Center for Energy and Environment of Chongqing, College of Materials Science and Engineering, Chongqing University Chongqing 400044 China ming.kong@cqu.edu.cn; Chongqing Key Laboratory of Vanadium-Titanium Metallurgy and New Materials, Chongqing University Chongqing 400044 PR China

## Abstract

Synergies between arsenic (As) and potassium (K) species in the deactivation of V_2_O_5_-WO_3_/TiO_2_ catalyst were investigated. Both arsenic oxide and potassium species presented a serious poisoning impact on catalyst activities, and the extent of poisoning of (As + K) was much stronger than their single superposition. The intrinsic reasons were explored and analyzed by N_2_ physisorption, XPS, H_2_-TPR, NH_3_-TPD, NH_3_-DRIFTS and *in situ* FTIR. Results indicated that BET surface area decreased due to the formation of a dense arsenic coating on the catalyst surface. V–OH active sites were destroyed by arsenic and As–OH acid sites were newly generated. After potassium species were added to arsenic-poisoned catalyst, K^+^ further neutralized the As–OH acid sites, and the amount and stability of both Lewis and BrØnsted acid sites decreased more greatly. Potassium also reacted with intermediate NH_2_^−^ when the temperature was elevated to higher than 250 °C, which resulted in more NH_3_ consumption and NH_3_-SCR reaction inhibition. The extent of deactivation was related to the potassium species when both poisons reacted on the catalyst, and the influence sequence followed AsKS < AsKN < AsKC. As_2_O_3_ + K_2_SO_4_ presented the weakest impact among these three poisoned catalysts due to the resistance of SO_4_^2−^ to arsenic.

## Abstract 2

Synergies between arsenic (As) and potassium (K) species in the deactivation of V_2_O_5_-WO_3_/TiO_2_ catalyst were investigated.

## Graphical abstract

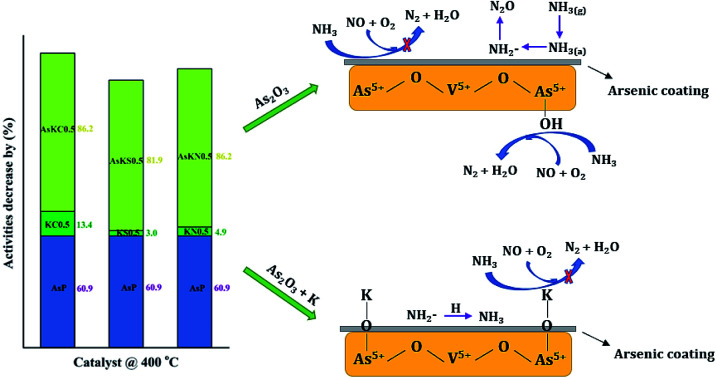

## 1. Introduction

As one of the major causes of acid rain, photochemical smog and PM2.5 pollution, nitrogen oxides (NO_*x*_), emitted from stationary and mobile sources, have attracted much attention in recent years. The selective catalytic reduction of NH_3_ (NH_3_-SCR) has been considered as one of the most economic and efficient technologies for controlling NO_*x*_ emission, and V_2_O_5_-WO_3_ (MoO_3_)/TiO_2_ is the most widely used catalyst at present.^1–4^ However, there are still some big problems that need to be solved, such as high working temperature, deactivation, ammonia oxidation and escape, and SO_2_ oxidation. Among these problems, deactivation is the restrictive factor for employing the catalyst, and alkali metals and arsenic poisoning are the two primary reasons responsible for it, based on previous studies.^5–8^ Therefore, if it is intended to extend the life span of the catalyst and improve its service efficiency, the mechanism of catalyst deactivation resulting from these poisons should be firstly controlled, and only in that case the corresponding solutions could probably to be put forward.

Alkali metals, always existing in the form of oxide and salt in flue gas, are the major components that deactivate SCR catalyst, and their effect on the traditional catalyst has been explored for many years.^6,9–14^ Previous researches consistently found that alkali metals in the main group IA (K and Na) present a greater extent of poisoning than the elements in the main group IIA (Ca and Mg). They usually deactivate V_2_O_5_-WO_3_(MoO_3_)/TiO_2_ catalysts by neutralizing the BrØnsted acid sites, decreasing the surface chemisorption of oxygen and reducing the reducibility of vanadium species. Moreover, the reaction state of the alkali metals on the catalyst also influences the mechanism of catalyst deactivation, which involves their existing phases and species.^15^ Arsenic is another poison for deactivating SCR catalyst. It usually exists as oxide As_2_O_3_ or its dimer As_4_O_6_ in flue gas.^16,17^ In general, the introduction of arsenic decreases the Lewis and BrØnsted acid sites on V_2_O_5_-WO_3_/TiO_2_ catalyst, and the less active As–OH is newly formed simultaneously.^18–21^ At the same time, arsenic improves catalyst reducibility and increases surface-active oxygen to strengthen catalyst oxidizability, which result in the promotion of NH_3_ oxidation and the decrease in N_2_ selectivity. All the above aspects cause the catalyst activity to decline. However, under the actual working conditions of V_2_O_5_-WO_3_/TiO_2_ catalyst, both alkali metals and arsenic in the flue gas interact with the catalyst active components on the catalyst surface during the NH_3_-SCR de-NO_*x*_ process. It results in the decline of catalyst activities as well as decrease in catalyst life span. Therefore, in order to solve the above problems, a possible mechanism of synergies between alkali metals and arsenic on V_2_O_5_-WO_3_/TiO_2_ catalyst should firstly be elucidated. But viewing the previous researches, it is found that none of them have considered their synergetic reactions.

Herein, potassium was selected as the representative alkali metal and three potassium species (KCl, K_2_SO_4_ and KNO_3_) were compared. Arsenic (As_2_O_3_) was loaded on commercial V_2_O_5_-WO_3_/TiO_2_ catalyst by vapor deposition and the sample with high arsenic content was selected for discussion. The catalyst properties were characterized by N_2_ physisorption, XPS, H_2_-TPR, NH_3_-TPD, NH_3_-DRIFTS and *in situ* FT-IR to clarify the synergies between arsenic and the different potassium species in the deactivation of V_2_O_5_-WO_3_/TiO_2_ catalyst.

## 2. Experimental

### 2.1 Catalyst preparation

Fresh V_2_O_5_-WO_3_/TiO_2_ catalyst was obtained from a catalyst company in southwest China, where the V_2_O_5_ is approximately 1 wt%, WO_3_ is about 5 wt% and TiO_2_ >90 wt%. As_2_O_3_-poisoned catalyst (denoted as ‘AsP’), prepared *via* vapor deposition in air at 400 °C, was selected and the As_2_O_3_ content was found to be 3.04 wt% by XRF measurement. Different potassium-poisoned catalysts were obtained by, respectively, impregnating the fresh catalyst into KCl, K_2_SO_4_ and KNO_3_ solution with different concentrations, and then the solutions were stirred at room temperature for 6 h, dried at 110 °C for 12 h and calcined for 5 h in air at 400 °C. These samples were denoted as KC*x*, KS*x* and KN*x*, where *x* is the potassium concentration in weight percent. The co-doped poisoned catalysts were prepared by impregnating AsP sample into different potassium solutions, and the obtained catalysts were marked as AsKC*x*, AsKS*x* and AsKN*x*, respectively. Samples with potassium loadings of 1.0 wt% were selected for characterization later.

### 2.2 Catalyst characterization

The element content in the catalyst was measured by PANalytical εpsilon3-XL X-ray fluorescence (XRF) spectrometer.

The BET surface area, pore size and pore volume of the samples were measured by N_2_ physisorption at 77 K using Micromeritics ASAP 2010 instrument.

Temperature-programmed desorption of NH_3_ (NH_3_-TPD) and temperature-programmed reduction of H_2_ (H_2_-TPR) experiments were conducted on a chemisorption analyzer (AutoChem II 2920, Micromeritics Instrument). In a typical NH_3_-TPD experiment, 100 mg of the sample was pretreated with He (50 ml min^−1^) for 1 h at 400 °C and then cooled down to 100 °C. Prior to the temperature-programmed desorption, the sample was exposed to a gas mixture of 5% NH_3_ in He (50 ml min^−1^) for 1 h and then purged with He until the TCD signal was stable. Finally, the sample was heated up to 500 °C (10 °C min^−1^) under a flow of helium (50 ml min^−1^) to record the NH_3_ TCD signal. For H_2_-TPR experiment, 100 mg of the sample was placed in one arm of a U-shaped quartz tube and then exposed to 4% H_2_ in Ar (50 ml min^−1^), and heated from 50 °C to 850 °C at a rate of 10 °C min^−1^.

X-ray photoelectron spectroscopy (XPS) profiles were acquired with a Thermo-Scientific system at room temperature using Al Kα radiation (1484.6 eV). The binding energy was referenced to the C 1s line at 284.6 eV, and peak deconvolution was performed using the Thermo Avantage v5.973 software.

NH_3_-DRIFTS (diffuse reflectance infrared Fourier transform spectroscopy) was recorded on a Thermo Scientific Nicolet iS50 spectrometer, which was equipped with a Polaris IR cell and an MCT detector cooled by liquid N_2_. Prior to the experiment, catalysts were firstly purged at 350 °C for 1 h under N_2_ gas (total flow rate 100 ml min^−1^), and the background spectrum at the desired temperature was collected. NH_3_ was adsorbed at 50 °C with 500 ppm NH_3_/N_2_ flow for 1 h, and then N_2_ flush for 1 h. Before scanning, the samples were kept for 30 min at the desired temperature and the DRIFT spectra were collected by accumulating 64 scans at a resolution of 4 cm^−1^.

### 2.3 Catalyst activity measurements

The catalyst activity measurements were conducted in a fixed-bed reactor, containing 200 mg sample of 40–60 mesh. The feed gas mixture contained 500 ppm NO, 500 ppm NH_3_, 5% O_2_, and He as the balance gas. The total gas flow rate was 500 ml min^−1^. The outlet NO_*x*_ concentration was monitored by a Thermo Electron Model 17C chemiluminescent NH_3_–NO_*x*_ gas analyzer, and the measurement was carried out between 200 and 500 °C at intervals of 50 °C. Each data acquisition was preceded by equilibration for 20 minutes. The catalytic activity was evaluated by NO_*x*_ conversion (%) according to the following equation:1
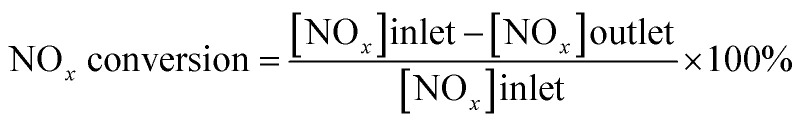


## 3. Results and discussion

### 3.1 Catalytic activity

**Fig. 1:**
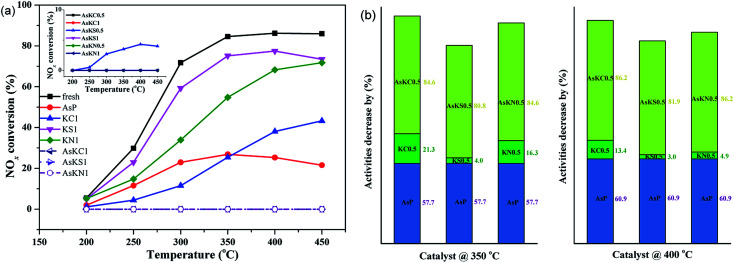
(a) NO_*x*_ conversion of fresh and poisoned commercial catalysts, (b) percent of activity decrease of fresh catalyst at 350 and 400 °C. Reaction condition: NO = NH_3_ = 500 ppm, O_2_ = 5%, total flow rate = 500 ml min^−1^, GHSV = 12 000 h^−1^.

Fig. 1(a) presents the NO_*x*_ conversion of fresh and poisoned catalysts under a GHSV of 12 000 h^−1^. When the poisons are added, the catalytic activity of the fresh catalyst is suppressed to different degrees. Arsenic oxide at 3.04 wt% exhibits serious poisonousness to the commercial catalyst, and only 25% of NO_*x*_ conversion is obtained at 350 °C. In the case of potassium-poisoned catalysts, the extent of deactivation is related to the potassium species and follows KCl > KNO_3_ > K_2_SO_4_. When both arsenic and potassium are loaded, NO_*x*_ conversion of all the samples is 0%, which means they are totally deactivated under the condition of 3.04 wt% As_2_O_3_ and 1.0 wt% potassium. To better understand the effect of potassium on arsenic-poisoned catalyst, the activity of arsenic-poisoned catalyst with different amounts of potassium was also examined, and the results are shown in the illustration in Fig. 1(a). Obviously, only AsKS 0.5 sample presents SCR activity, and only 4.3% NO_*x*_ conversion is obtained at 400 °C. For other samples, the NO_*x*_ conversion is still 0%. Decreasing the potassium content from 1 wt% to 0.5 wt% does not relieve catalyst deactivation. It seems that both the potassium species and content are the major factors determining the catalytic activity under the condition of potassium and arsenic co-existing on commercial SCR catalyst. The introduction of SO_4_^2−^ from K_2_SO_4_ is responsible for its activity recovery, as it is reported that SO_4_^2−^ exhibits the ability to resist arsenic to a certain extent.^20^

Notably, it can be seen from Fig. 1(b) that in comparison with the deactivation degree of a single-component poisoned catalyst, the synergetic effect of arsenic and potassium is much stronger than their superposition (poisoning extent (As + K) > As + K). It means that arsenic and potassium species are not separately playing a role in catalyst deactivation, but there are some interactions occurring between them.

### 3.2 Textural properties of the catalysts

**Fig. 2:**
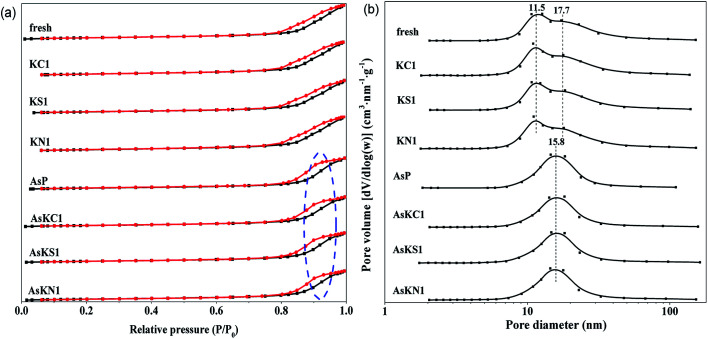
(a) N_2_ physisorption isotherms and (b) pore size distribution of fresh and poisoned catalysts.

N_2_ physisorption isotherms and pore size distribution of all the catalysts are provided in Fig. 2, and the corresponding textural data are summarized in Table 1. The fresh and potassium-poisoned catalysts present similar isotherms that belong to type IV with H1 hysteresis loops at high relative pressure range (*P*/*P*_0_), which is characteristic of mesoporous materials. However, a totally different desorption branch, belonging to H2 hysteresis loop, appears in arsenic-poisoned catalysts. It is resulted from the channel plugging ascribed to arsenic deposition. Their pore size distribution is also totally different from that of the other catalysts. They yield a pore diameter distribution centered at approximately 15.8 nm, while the others yield at about 11.5 nm with a shoulder peak at 17.7 nm. It is indicative that arsenic deposition brings about changes to the catalyst pores. This result can also be proved by the data in Table 1. It can be seen that the BET surface area and total pore volume of the fresh catalyst decrease, and the average pore size increases after arsenic introduction. Although an irregular trend concerning the BET surface area and pore size is seen in potassium-poisoned catalysts, in the case of arsenic-poisoned catalysts, adding potassium distinctly decreases their BET surface area and the pore size increases. Therefore, arsenic is the dominant factor affecting the textural properties of commercial SCR catalysts when arsenic and potassium coexist.

**Table 1:** The BET surface area, pore volume and pore size of the catalysts

Catalysts	BET surface area (m^2^ g^−1^)	Total pore volume (cm^3^ g^−1^)	Pore size (nm)
Fresh	55.8	0.21	14.84
AsP	52.9	0.20	15.17
KC1	58.0	0.20	14.13
KS1	54.2	0.20	14.66
KN1	58.5	0.21	14.27
AsKC1	48.9	0.20	16.14
AsKS1	47.1	0.19	16.15
AsKN1	49.2	0.19	15.37

### 3.3 Chemical state of elements in the catalysts (XPS)

**Fig. 3:**
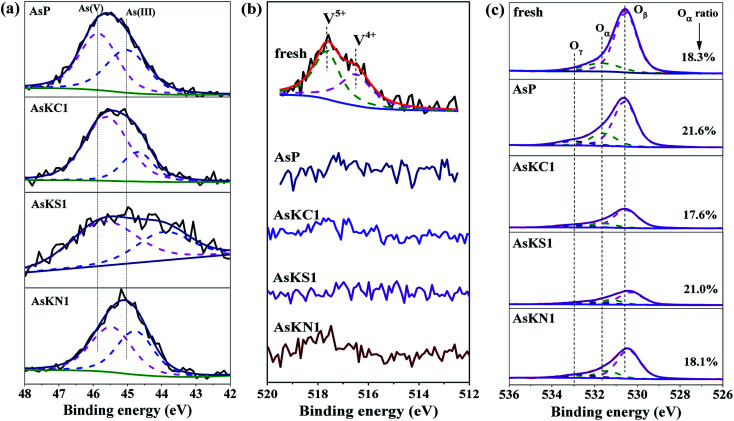
XPS spectra of (a) As 3d, (b) V 2p and (c) O 1s for the fresh and poisoned catalysts.

The XPS measurement was conducted to explore the synergetic effect of arsenic and potassium on the content and chemical environments of active elements on the surface of fresh and poisoned catalysts. However, the detection depth is only 2–3 nm for oxides, so the XPS results could only reflect the state of the external surface.^22^ The XPS spectra with the corresponding deconvolution results of As, V and O elements are shown in Fig. 3. The As 3d spectra of arsenic-containing samples are presented in Fig. 3(a); two peaks can be fitted in each curve. Referring to previous reports, the peaks in AsP sample at 45.0 eV and 45.9 eV can be attributed to As^3+^ and As^5+^, respectively,^23,24^ and more than half of the original As^3+^ is oxidized to As^5+^. After potassium doping, two peaks can be observed in each sample, but both peaks are shifted to the lower binding energy, especially for AsKS1 catalyst. It means that the potassium species enters into the lattice of the arsenic species and influences its chemical environment. KCl and KNO_3_ exhibit a similar impact on arsenic-poisoned catalyst for both As^3+^ and As^5+^, but K_2_SO_4_ shows a stronger influence on As^3+^. Other than the interactions between K and arsenic species, their lattice structure is also changed by SO_4_^2−^.

Fig. 3(b) shows the V 2p spectra of all catalysts, and two peaks can be fitted after deconvolution in the fresh catalyst. The peaks centered at 517.7 eV and 516.5 eV can be assigned to V^5+^ and V^4+^, respectively, and both the valent V species are present on the catalyst surface.^25,26^ However, for AsP sample, no V peak signal is detected, and no changes are observed after potassium loading. This may be related with the fact that the arsenic species cover the catalyst surface and prevent V signal detections. The coverage by arsenic also prevents reactions between the reduction agent NH_3_ and vanadia active sites, which causes an extremely severe deactivation. Therefore, in the research, the formation of arsenic coating is one of the major causes for catalyst deactivation when it co-exists with potassium. This result is also in accordance with the catalytic activities above.

The XPS spectra of O 1s for fresh and poisoned catalysts were also investigated and the results are exhibited in Fig. 3(c). All spectra are fitted into three peaks centered at, approximately, 530.6, 531.6 and 532.7 eV, which can be, respectively, assigned to lattice oxygen in metal oxides (O_β_), surface chemisorbed oxygen (O_α_) and hydroxyl species and/or adsorbed water species (O_γ_).^6,27–29^ The O_α_ ratio is also calculated because the chemisorbed oxygen species is more active than lattice oxygen due to its higher mobility, which plays a crucial role in the SCR process.^30,31^ It is clear that the O_α_ fraction of the fresh catalyst increases from 18.3% to 21.6% after the addition of arsenic. This result is coincident with the results of previous literatures that arsenic oxide is positive for improving surface chemisorbed oxygen of V_2_O_5_-WO_3_/TiO_2_ catalyst.^20,21^ When arsenic and potassium simultaneously exist in the catalyst, not only does the peak strength weaken, but the O_α_ ratio also decreases. Potassium decreases the surface chemisorbed oxygen of arsenic-poisoned catalyst, and the downtrend follows KCl > KNO_3_ > K_2_SO_4_, which is in agreement with the activity results. As a consequence, the drop in O_α_ ratio, resulting from potassium doping, is another reason responsible for the enhanced deactivation of arsenic and potassium co-poisoned catalysts.

### 3.4 Reducibility of the catalyst (H_2_-TPR)

**Fig. 4:**
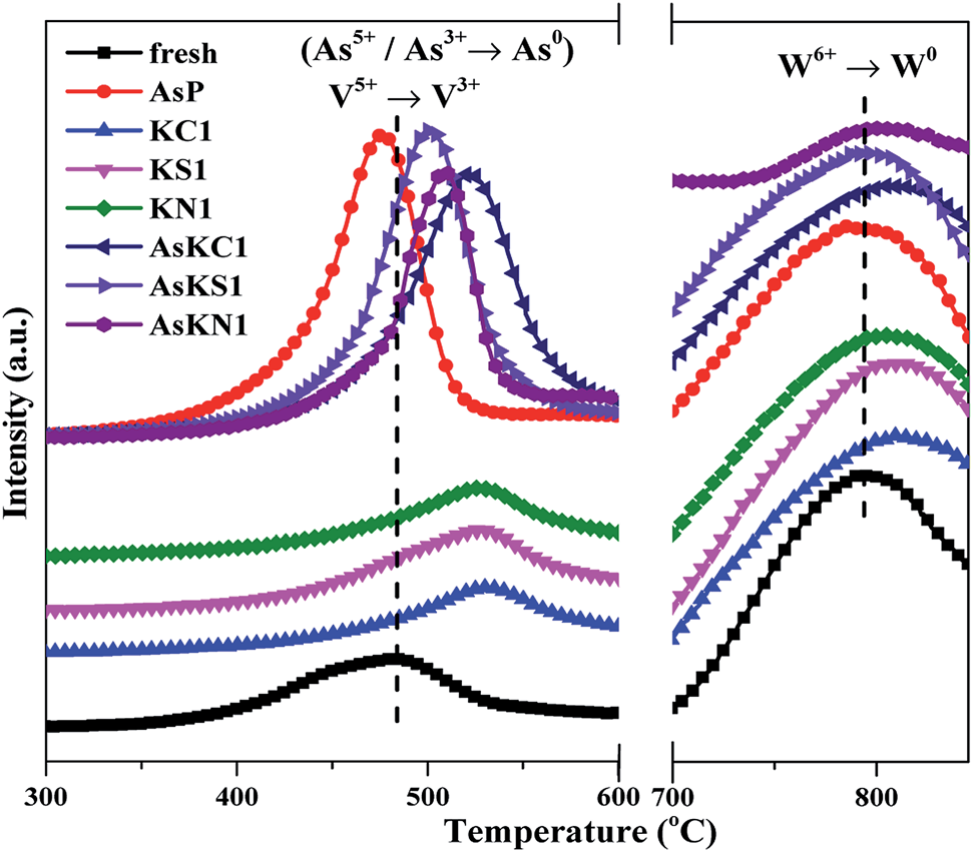
H_2_-TPR profiles of fresh and poisoned catalysts. Measurement condition: 5% H_2_/He, total flow rate 50 ml min^−1^.

Researches have proven that redox ability is one of the key properties in the SCR process. Therefore, H_2_-TPR measurement was conducted, and the profiles are shown in Fig. 4. Two main reduction peaks are exhibited in the fresh sample, which can be attributed to the reduction of V^5+^ (∼481 °C) and W^6+^ (∼797 °C).^32–34^ It is quite clear that both the reduction peaks are shifted to higher temperature for potassium-poisoned catalysts, meaning that the reducibility of the pristine catalyst is weakened by the potassium species. In comparison, the effect of potassium on tungsten oxide is much weaker than its effect on vanadium oxide. However, an obviously larger H_2_ consumption peak corresponding to As^5+^/As^3+^ reduction is observed in AsP catalyst and covers the V^5+^ reduction peak. The reduction peak at lower temperature for AsP catalyst indicates that arsenic can improve catalyst reducibility, but the enhancement promotes N_2_O generation instead of contributing to NO reduction (Fig. S1†). As expected, the reduction temperature of co-poisoned catalysts is higher than that of fresh and AsP catalysts, but lower than that of potassium-poisoned catalysts. The sequence follows AsKS1 < AsKN1 < AsKC1, which is consistent with the extent of deactivation of potassium-poisoned catalysts. It manifests that the reducibility of co-poisoned catalysts is decided by both poisons, that is arsenic improves N_2_O formation and potassium weakens NO reduction. Both aspects together result in the lower catalytic activities and more drastic deactivation.

### 3.5 Surface acidity (NH_3_-TPD and NH_3_-DRIFTS)

**Fig. 5:**
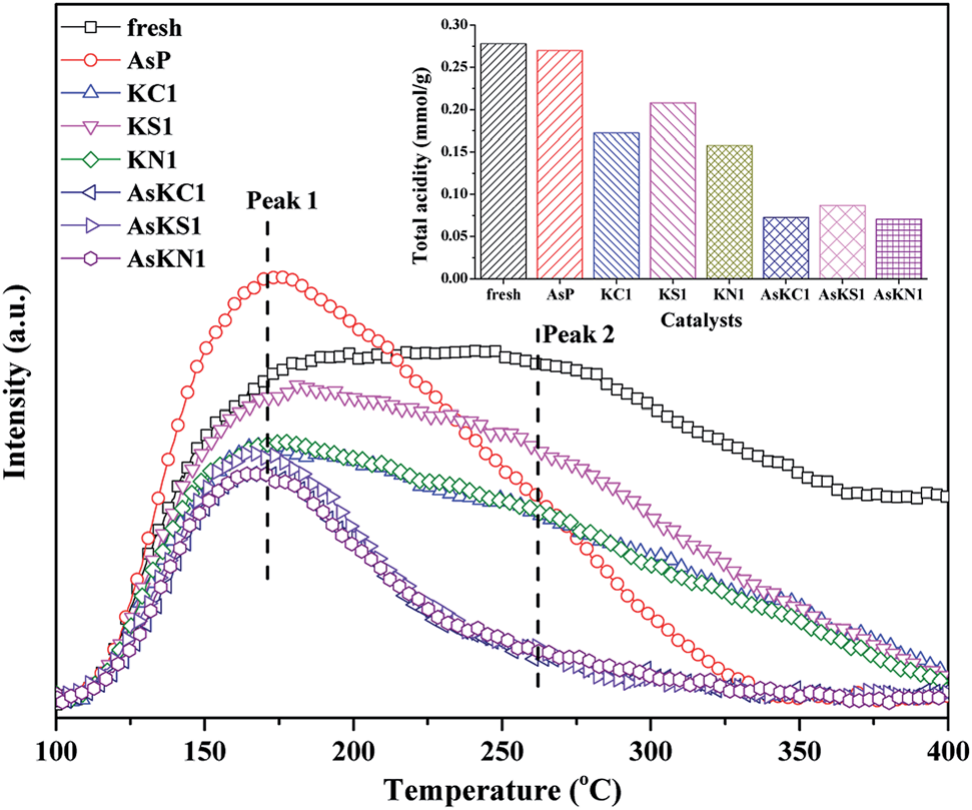
NH_3_-TPD profiles of fresh and poisoned catalysts and the corresponding amount of total acidity.

Fig. 5 shows the NH_3_-TPD profiles of fresh and poisoned catalysts, and the relative amount of total acidity is also presented in the illustration. The fresh catalyst shows the largest amount of acidity and the signal still holds until 400 °C. Potassium doping decreases the acid sites of the fresh catalyst and the amount of surface acidity follows KS1 > KC1 > KN1. Each curve can be divided into two peaks centered at around 170 °C (peak 1) and 260 °C (peak 2), which, respectively, correspond to the weakly and strongly bound ammonia on the BrØnsted and Lewis acid sites.^35^ For AsP catalyst, the weak acid sites increase whereas the strong acid sites decrease greatly. The increase in weak acid sites can be assigned to the newly generated As–OH.^20^ When arsenic and potassium are loaded on the catalyst simultaneously, the amount of both weak and strong acid sites decreases. Although arsenic enhances the weak acid sites, potassium introduction further neutralizes the new acid sites. AsKS1 still shows the largest amount of acidity among these three catalysts due to the existence of SO_4_^2−^, and it provides some new acid sites. Besides, compared to the original acid sites in the fresh catalyst, potassium more easily neutralizes the newly formed As–OH and arsenic further promotes the negative effect of potassium on the Lewis acid sites.

**Fig. 6:**
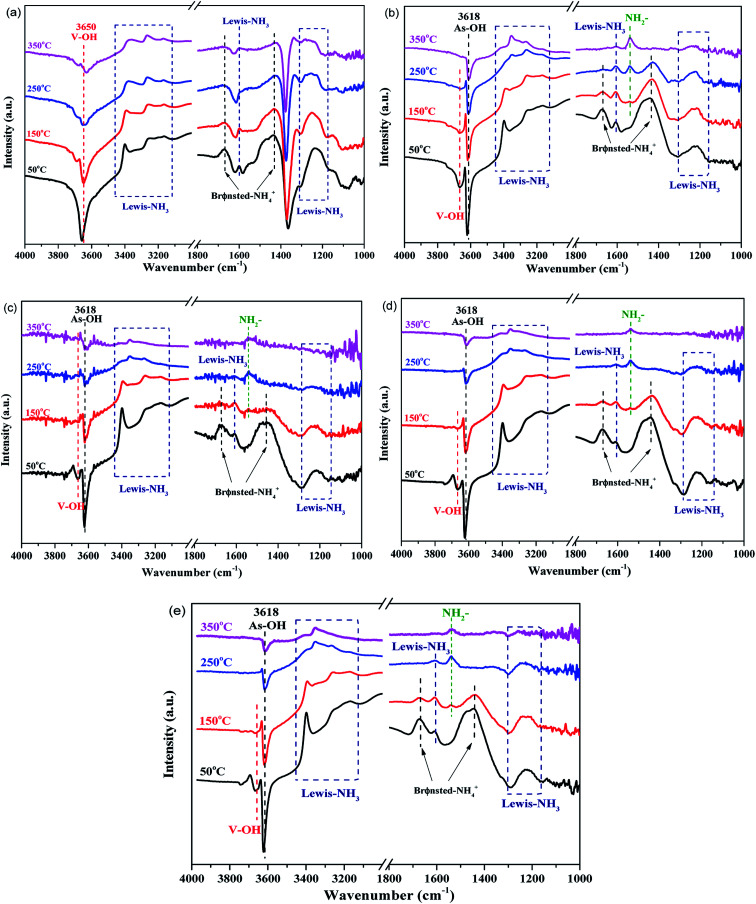
NH_3_-DRIFTS spectra of (a) fresh, (b) AsP, (c) AsKC1, (d) AsKS1 and (e) AsKN1 catalysts at 50–350 °C.

NH_3_-DRIFTS measurements were employed to further explore the NH_3_ adsorption behavior on the acid sites of different catalysts. As shown in Fig. 6, the spectra are obtained upon NH_3_ adsorption at 50 °C and then evacuation at elevating temperature. It can be obviously seen that, on AsP sample, the major NH_3_ adsorption sites of the fresh sample, V–OH (3650 cm^−1^),^36^ Lewis acid sites (3100–3450 cm^−1^, 1150–1300 cm^−1^, 1600 cm^−1^) and BrØnsted acid sites (1670 cm^−1^, 1430 cm^−1^),^15,21^ diminish remarkably and almost vanish with rise in temperature. It means the catalyst surface acidity and stability are impaired by arsenic. Meanwhile, two new sites are generated – As–OH (3618 cm^−1^),^37^ ascribed to V replacement by As, and NH_2_^−^ (1537 cm^−1^),^3,38,39^ ascribed to NH_3_ oxidation. It is one of the evidences for AsP catalyst deactivation due to the reduction of N_2_ selectivity and lower reactivity of As–OH. When potassium species are loaded afterwards, NH_3_ adsorption sites further diminish more remarkably because of the intrinsic influence of potassium on the acid sites of the fresh catalyst. Moreover, it should be noted that, the strength of the As–OH peak also weakens after potassium introduction and the extent of impairment is more drastic as temperature rises compared to the results of AsP sample. This phenomenon can be attributed to the further reaction between potassium and As–OH in which H is replaced by K and As–OK may be finally generated. As can be seen in AsP catalyst, more NH_2_^−^ are generated with increase in temperature. However, for the co-poisoned catalysts, the peak strength decreases instead when temperature rises from 250 °C to 350 °C. Potassium further reacts with the intermediate of NH_3_ oxidation at higher temperature (>250 °C), and then N_2_O generation is inhibited. The three potassium species present a similar performance (as shown in Fig. S1†). The probable reaction procedure can be described as follows (from eqn (2)–(4)):

2 NH_3_ (g) → NH_3_ (ads)

3 NH_3_ (ads) + O (surf) → NH_2_^−^ + –OH

4 NH_2_^−^ + –OH + K → NH_3_ + –O–K

### 3.6 *In situ* studies of the reaction process (*in situ* FT-IR) and poisoning mechanism

**Fig. 7:**
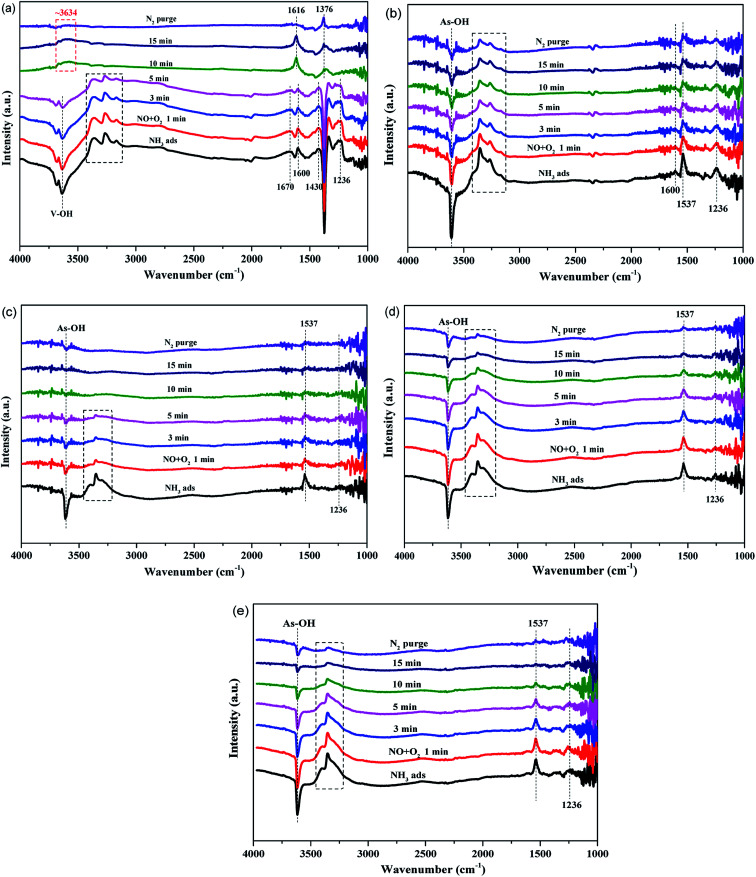
*In situ* FT-IR spectra of (a) fresh, (b) AsP, (c) AsKC1, (d) AsKS1 and (e) AsKN1 catalysts pretreated with 500 ppm NH_3_ for 1 h, and followed by exposure to 500 ppm NO and 5% O_2_ at 350 °C.

To clarify the changes in different catalysts during the SCR process, *in situ* FT-IR measurement was carried out at 350 °C. NO + O_2_ were introduced into the reaction unit after NH_3_ adsorption, and the results are presented in Fig. 7. In the spectra of the fresh catalyst, the characteristic peaks of NH_3_ adsorption gradually disappear as NO + O_2_ are continuously introduced and some new surface species appear after 10 min. These newly generated species are the products or intermediates during the SCR reaction, which can be respectively, assigned to the new OH groups bonded to the more oxidized V species (∼3634 cm^−1^), H_2_O (1616 cm^−1^) and NH_4_NO_3_ (1376 cm^−1^).^39–41^ When purged by N_2_ later on, these three peaks, as expected, weaken or vanish. However, for AsP catalyst, all the original characteristic peaks of NH_3_ adsorption decrease slightly and hold on until 15 min, which means they hardly react with NO and O_2_. This is also one of the major reasons for the deactivation of arsenic-poisoned catalyst. Moreover, the slightly weakened As–OH peak indicates that it participates in the SCR reaction but has lower reactivity. When (As + K) exist simultaneously in this system, the strength of all the original peaks decreases more intensively as reaction progresses. Potassium accelerates the degree of deactivation of arsenic-poisoned catalyst. Besides, it can be observed that the active sites in AsKC1 catalyst disappear first and in AsKS1 the last. It is because of the resistance of SO_4_^2−^ to arsenic poisoning. This result is also consistent with the activity measurement. Based on the above analysis, it can be concluded that different potassium species present different impacts on arsenic-poisoned catalyst, and the sequence follows KS < KN < KC.

**Fig. 8:**
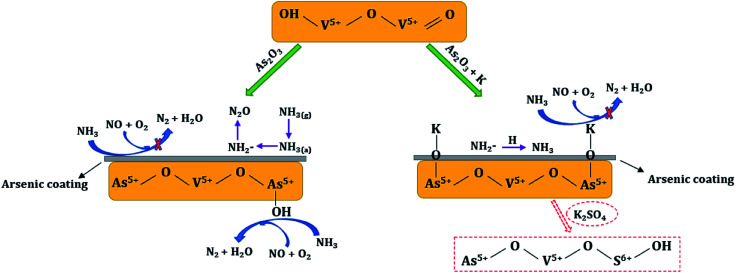
Mechanism of arsenic and potassium poisoning.

According to the above analysis, the possible synergetic mechanisms between arsenic oxide and potassium species in the deactivation of commercial V_2_O_5_-WO_3_/TiO_2_ catalyst are proposed. As shown in Fig. 8, with As_2_O_3_ introduction an arsenic coating is generated on the catalyst surface, and NH_3_ can hardly be adsorbed and activated to reduce NO, but is oxidized to generate NH_2_^−^ and N_2_O. Meanwhile, the less reactive As–OH is newly formed, and it also participates in the SCR reaction. When potassium species are loaded afterwards, H on the newly generated As–OH is further replaced by K to form As–O–K. The dissociative H^+^ then combines with NH_2_^−^ to form NH_3_. As a result, the co-existence of arsenic and potassium makes the SCR reaction more difficult. Specially, SO_4_^2−^ in the K_2_SO_4_ provides some new acid sites, which alleviates catalyst deactivation to a certain extent.

## 4. Conclusions

Arsenic and potassium species presented a serious poisoning impact on commercial V_2_O_5_-WO_3_/TiO_2_ catalyst during SCR process. A series of activity and property characterizations were carried out on fresh and poisoned catalysts in the research. Results indicated that the extent of poisoning of (As + K) is much stronger than their single superposition. Arsenic is the major cause that seriously changes the textural properties of poisoned catalyst, such as BET surface area and pore size distribution. It also greatly improves catalyst oxidizability, but this promotion is beneficial for NH_3_ oxidization instead of the SCR reaction. In addition, potassium inhibits catalyst redox ability, which makes catalyst deactivation more severe. On the other hand, V–OH active sites are destroyed by As and the less reactive As–OH is newly formed. Potassium introduction afterwards further neutralizes the As–OH acid sites, which is another reason for the much stronger deactivation of (As + K)-poisoned catalysts. Besides, the amount and stability of both the Lewis and BrØnsted acid sites also decrease more greatly in the (As + K) system. It is also found that potassium reacts with NH_2_^−^ during the NH_3_ oxidization process at temperature higher than 250 °C. During the SCR process, other than the influence of arsenic, the potassium species also exhibit different effects and the sequence follows AsKS < AsKN < AsKC due to the resistance of SO_4_^2−^ to arsenic.

Based on the above results, the synergies between arsenic and potassium species on the deactivation of V_2_O_5_-WO_3_/TiO_2_ catalyst are finally proposed: NH_3_ can hardly be adsorbed on V–OH because of the formation of arsenic coating and As destruction, and it is oxidized to N_2_O at high temperature (350 °C). The newly generated As–OH can participate in NO reduction, but it is less reactive. The introduction of potassium further neutralizes the As–OH acid sites to form As–O–K and the dissociative H^+^ replaced by K combines with NH_2_^−^ (coming from NH_3_ oxidization) to inhibit N_2_O generation. Additionally, in the As_2_O_3_ + K_2_SO_4_ system, SO_4_^2−^ provides some new acid sites, which ensures less deactivation of AsKS catalyst.

## Conflicts of interest

There are no conflicts to declare.

## Supplementary Material

RA-009-C9RA07545C-s001
