# Supplementary material for: New insights into the deactivation mechanism of V2O5-WO3/TiO2 catalyst during selective catalytic reduction of NO with NH3: synergies between arsenic and potassium species
Source: RSC Adv. 2019 Nov 19;9(65):37724–32. doi: 10.1039/c9ra07545c (PMC9075767; doi:10.1039/c9ra07545c)
Supplement: RA-009-C9RA07545C-s001 [file RA-009-C9RA07545C-s001.pdf]

Electronic Supplementary Material (ESI) for RSC Advances.
This journal is © The Royal Society of Chemistry 2019

Supporting Information

New insights into the deactivation mechanism of V_2O_5-WO_3/TiO_2 catalyst during selective catalytic reduction of NO with NH_3: Synergies between arsenic and potassium species

Lin Li [a, b], Lin Chen [a, b], Ming Kong [a, b *], Qingcai Liu [a, b], Shan Ren [a, b]

[a] *Engineering Research Center for Energy and Environment of Chongqing, College of Materials Science and Engineering, Chongqing University, Chongqing 400044, China*

[b] *Chongqing Key Laboratory of Vanadium-Titanium Metallurgy and New Materials, Chongqing University, Chongqing 400044, PR China*

a, b) Corresponding Author: Ming Kong (M. Kong), e-mail: ming.kong@cqu.edu.cn*

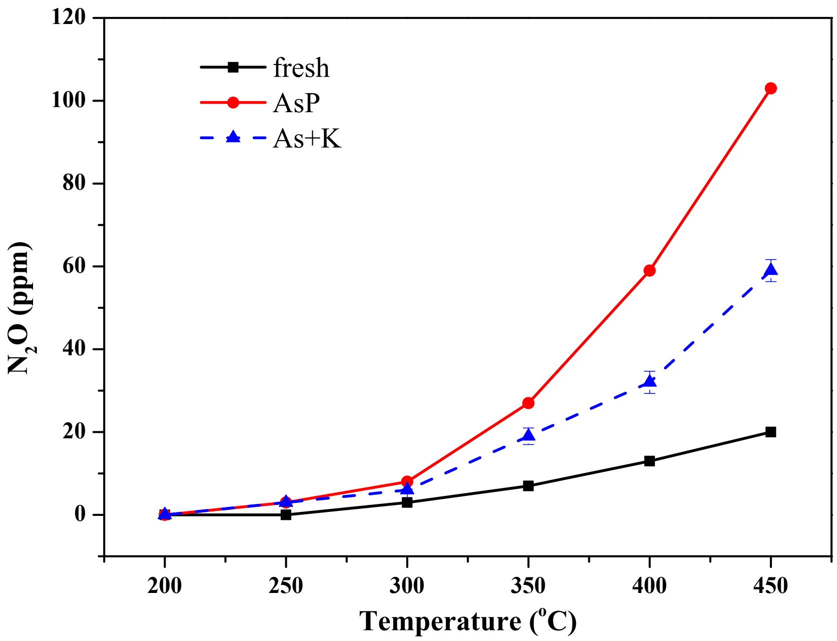

Fig. S1 Concentration of outlet N_2O

Reaction condition: NO=NH_3=500 ppm, O_2=5%, total flow rate=500 ml/min, GHSV=12,000 h^{-1}.
